# Role of the score for the targeting of atrial fibrillation (STAF) combined with D-dimer in screening ischemic stroke patients with atrial fibrillation

**DOI:** 10.5937/jomb0-44255

**Published:** 2024-01-25

**Authors:** Libin Liu, Peikai Xie, Peipei Zhu, Wenyan Zhuo, Anding Xu

**Affiliations:** 1 Zhuhai Hospital Affiliated with Jinan University, Department of Neurology, Zhuhai, China; 2 Zhuhai Hospital Affiliated with Jinan University, Department of Ultrasound Imaging, Zhuhai, China; 3 First Affiliated Hospital of Jinan University, Department of Neurology, Guangzhou, China

**Keywords:** STAF, D-dimer, atrial fibrillation, ischemic stroke, combined test, STAF, D-dimer, atrijalna fibrilacija, ishemijski moždani udar, kombinovani test

## Abstract

**Background:**

We aim to explore the effect of the score for the targeting of atrial fibrillation (STAF) combined with the serum D-dimer (DD) level in screening acute ischemic stroke patients with atrial fibrillation (AF).

**Methods:**

This study is a retrospective case observation study. This study consecutively selected patients with acute ischemic stroke who were hospitalized in the Department of Neurology at Zhuhai Hospital Affiliated with Jinan University from February 2019 to February 2021. Venous blood was drawn from all patients within 24 hours of hospitalization for DD detection. In accordance with the medical records, the patients were classified into an AF group and a non-AF group and were scored according to the STAF standard. A combined test method was used to estimate the diagnostic screening value of the STAF combined with the DD value for acute ischemic stroke patients with AF.

## Abstract (sr)

**Uvod:**

Cilj je bio da se istraži efekat skora za ciljanje atrijalne fibrilacije (STAF) u kombinaciji sa nivoom serumskog D-dimera (DD) u skriningu pacijenata sa akutnim ishemijskim moždanim udarom sa atrijalnom fibrilacijom (AF).

**Metode:**

Ova studija je retrospektivna studija posmatranja slučaja. Ova studija je uzastopno birala pacijente sa akutnim ishemijskim moždanim udarom koji su bili hospitalizovani na Odeljenju za neurologiju u bolnici Zhuhai pri Univerzitetu Jinan od februara 2019. do februara 2021. Venska krv je uzeta svim pacijentima u roku od 24 sata od hospitalizacije radi otkrivanja DD. U skladu sa medicinskom dokumentacijom, pacijenti su klasifikovani u AF grupu i ne-AF grupu i bodovani su prema STAF standardu. Kombinovana metoda ispitivanja je korišćena za procenu dijagnostičke skrining vrednosti STAF u kombinaciji sa vredno u DD za pacijente sa akutnim ishemijskim moždanim udarom sa AF.

**Rezultati:**

Uključeno je ukupno 480 pacijenata, od kojih je 73 bolesnika (15,2%) imalo AF, a 42 pacijenta paroksizmalnu AF. Dijagnostička osetljivost STAF ≥ 5 za pacijente sa AF bila je 78%, a specifičnost 91%. Dijagnostička osetljivost DD ≥ 826,5 ng/mL za AF bila je 70%, a specifičnost 76%. Dijagnostička osetljivost dva indikatora kombinovana za AF bila je 86%, a specifičnost 97%.

**Zaključak:**

STAF u kombinaciji sa nivoom DD u serumu ima dobru dijagnostičku vrednost u skriningu pacijenata sa akutnim ishemijskim moždanim udarom sa AF.

## Introduction

Ischemic stroke can be classified into two major categories: cardiogenic stroke and non cardiogenic stroke, and the secondary prevention plans of the two are different. Atrial fibrillation (AF) is the most common risk factor for cardiogenic stroke. Studies have reported that approximately one-fourth to one-third of ischemic stroke patients have AF [1] [2]. According to the frequency of attacks, AF is divided into paroxysmal, persistent, or permanent AF. All types of AF significantly increase the risk of stroke. Among them, paroxysmal AF (PAF) accounts for approximately 60% of all AF. However, the current diagnosis rate of PAF is low because its diagnosis depends on multiple electrocardiography (ECG) examinations, ambulatory ECG, continuous ECG monitoring, and other timeconsuming and labor-intensive examinations [3]. A reliable preliminary screening method to screen suspected or high-risk PAF patients and guide further targeted examinations to define the presence of PAF will significantly increase the diagnosis rate of PAF.

Currently, there is no recognized highly effective screening method. The score for the targeting of atrial fibrillation (STAF) is a scoring system proposed by Suissa et al. [4] to screen for the existence of AF in patients with ischemic stroke, and it has a significant screening value, especially for PAF [5]. Table 1 shows the detailed scoring items. Subsequently, the STAF score has been widely used in clinical practice, although some studies have shown that its effectiveness is limited [6] [7]. The serum biochemical index D-dimer (DD) is a terminal degradation product of cross-linked fibrin hydrolyzed by plasmin. It is significantly increased during thrombosis and secondary fibrinolysis. Acute ischemic stroke patients have demonstrated activation of the coagulation and fibrinolysis system, and the plasma DD level of these patients may be elevated. Many studies have shown that DD is markedly increased in AF patients [8] [9], especially when complicated with cerebral embolism [10], and its degree of increase is significantly correlated with the risk of cerebral embolism [11] [12].

**Table 1:** Criteria and scoring for the STAF. NIHSS National Institute of Health Stroke Scale

Criteria		Points
Age, y	<62	2
	62	0
Baseline NIHSS score	≥8	1
	<8	0
Left atrial dilatation	Yes	2
	No	0
Vascular etiology	Yes	0
	No	3
**Total**		**0 to 8**

Currently, using a single method to screen for the existence of AF in ischemic stroke patients has limitations. To increase the diagnostic exactitude for AF, a combined test method should be employed. Both the STAF and DD are indicators that are easily available in the early stage of stroke in inpatients. To enhance the diagnostic rate of AF, this study used the receiver operating characteristic (ROC) curve and a combined test method to estimate the role of STAF combined with DD level in screening for AF.

## Materials and methods

This study is a retrospective case observation study. It was identified by the ethics committee of Zhuhai Hospital affiliated with Jinan University. Because all patients had been discharged from the hospital, no informed consent form was signed. However, the patients' personal information was concealed to protect their privacy. Acute ischemic stroke patients hospitalized in the Department of Neurology of Zhuhai Hospital Affiliated with Jinan University from February 2019 to February 2021 were consecutively enrolled.

Inclusion criteria: (1) Patients meet the diagnostic criteria of ischemic stroke in the 2018 Chinese Guidelines for the Diagnosis and Treatment of Acute Ischemic Stroke; (2) Disease onset time 7 days; (3) First acute ischemic stroke attack.

Exclusion criteria: (1) Aged<18 years; (2) Diagnosed with hemorrhagic stroke; (3) The presence of malignant tumors, thrombosis, or embolic diseases of other organs; (4) Patients undergoing intravenous or intra-arterial thrombolysis.

The baseline data of all patients, such as sex, age, previous medical history, personal history, and initial National Institute of Health Stroke Scale (NIHSS) score, as well as relevant laboratory tests and imaging examination results completed during hospitalization, including serum DD, fasting blood glucose, glycosylated hemoglobin, triglycerides, total cholesterol, low-density lipoprotein cholesterol, regular ECG, 24-hour Holter ECG, transthoracic echocardiography and transesophageal echocardiography, were collected and recorded.

The DD level was tested by the laboratory of our hospital. Four milliliters of fasting cubital venous blood was drawn from all patients on the morning after admission and submitted to the laboratory. The blood was treated with sodium citrate for anticoagulation and centrifuged at 3000 r/min for 10 minutes to separate the serum. DD was detected by the immuneturbidimetric method using an automatic blood coagulation analyzer and supporting kit (SYS-MEX, Japan).

All patients underwent STAF assessment in strict accordance with the standard [4]. Table 1 shows the detailed scoring items. Among them, determination of left atrial dilatation was based on the standard of a left atrial diameter of greater than 35mm measured by transthoracic echocardiography as proposed by the American Society of Ultrasound. The vascular etiology was defined according to the Trial of ORG 10172 in Acute Stroke Treatment (TOAST) etiology classification. Large artery atherosclerosis or small artery occlusion stroke types were classified as having a vascular etiology; otherwise, stroke was classified as not having a vascular etiology. The diagnosis of AF was based on the patient's previous history, cardiac auscultation, regular ECG, ambulatory ECG, and ECG monitoring and was classified persistent AF or PAF. All enrolled patients were classified into an AF group and a non-AF group, and the differences in the STAF and serum DD level were contrasted between the two groups.

Statistical analysis of all data was performed using SPSS19.0 statistical software. The means were compared between the two groups using a t test. The rank sum test was used to analyze data with abnormal distribution and uneven variance. The ROC curve was employed to assess the accuracy of AF diagnosis and to measure the sensitivity and specificity. A combined test method was used for AF diagnosis by combining two indicators.

## Results

A total of 480 patients were enrolled, including 298 males, accounting for 62.08%, and 182 females, accounting for 37.92%. The patients were aged 25 to 87 years with an average age of 64.76±11.83 years. There were 73 patients with AF (15.2%), including 42 patients with PAF, and 31 patients with persistent AF. Among them, there were 18 cases of rheumatic heart disease, 23 cases of coronary atherosclerotic heart disease, 14 cases of hypertensive heart disease, and 18 cases of simple AF; 30 patients were diagnosed with AF previously, and 43 patients were diagnosed after comprehensive examinations after admission.

Both the STAF and DD value showed a skewed distribution and thus are represented by the median and interquartile range as shown in Table 2. The Mann-Whitney test for two-sample comparison was used to compare the STAF between the AF group and the non-AF group. The resulting values (*Z*=8.12 and *P*=0.000) indicated that the STAF was obviously higher in the AF group (mean rank of 280.73) than the non-AF group (mean rank of 145.33). When the DD level was compared between the two groups, the DD level of the AF group (mean rank of 235.50) was significantly higher (*Z*=4.85 and *P*=0.000) than the non-AF group (mean rank of 150.64).

**Table 2:** STAF and DD value of the AF and non-AF groups.

	AF group	Non-AF group
STAF	5(1)	2 (1.5)
DD (ng/mL)	880.93 (863.92)	366.09 (500.62)

### ROC curve of the STAF for the diagnosis of AF

**Figure 1:**
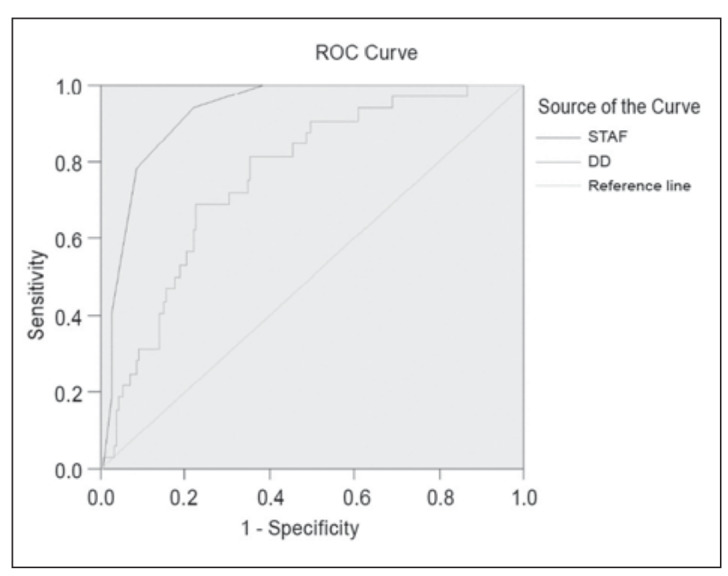
ROC curves of AF diagnosis based on STAF and DD. The area under the ROC curve (AUC) of STAF was 0.927. The AUC of DD was 0.761.

SPSS 19.0 statistical software was used to determine that the area under the ROC curve (AUC) of the STAF for the diagnosis of AF was 0.927 (Figure 1). According to the cut-off value, the diagnostic sensitivity of a STAF≥5 for AF was 78%, and the specificity was 91%.

### ROC curve of DD for the diagnosis of AF

The AUC of the DD level for the diagnosis of AF was 0.761 (Figure 1), indicating a moderate diagnostic value. According to the cut-off value, the sensitivity of DD≥826.5 ng/mL for diagnosing AF was 70%, the specificity was 76%.

### Combination of the STAF and DD level for the diagnosis of AF

The ROC curves showed that the sensitivity of using a STAF ≥ 5 or DD ≥ 826.5 ng/mL alone to diagnose AF was not significantly high, and the specificity of the latter was not good. To improve the efficiency of diagnosis, a combined test can be used to enhance the sensitivity and specificity. Combined tests include parallel and serial diagnostic tests. If diagnosis of AF can be achieved by either the STAF or DD level with a value higher than the corresponding cutoff value, these two diagnostic methods are parallel diagnostic tests, which can improve the sensitivity of the method. As shown in Table 3, the combined assessment indexes were as follows: sensitivity = 63/73×100%=86% and specificity=290/407 × 100%=71%.

**Table 3:** Parallel diagnostic test combining the STAF and DD level for the diagnosis of AF.

STAF and DD value	AF patients	Non-AF patients	Total
STAF≥5 and/or DD≥ 826.5 ng/mL	63	117	180
STAF<5 and DD<826.5 ng/mL	10	290	300
**Total**	**73**	**407**	**480**

If both the STAF and DD level are required to be greater than their respective cut-off values to diagnose AF, these two diagnostic methods are considered serial diagnostic tests, which can enhance the specificity of the method. As shown in Table 4, the combined assessment indexes were as follows: sensitivity=45/73×100%=62% and specificity=395/407 ×100%=97%.

**Table 4:** Serial diagnostic test combining the STAF and DD level for the diagnosis of AF.

STAF and DD value	AF patients	Non-AF patients	Total
STAF≥5 and DD≥ 826.5 ng/mL	45	12	57
STAF<5 and/or DD < 826.5 ng/mL	28	395	423
**Total**	**73**	**407**	**480**

## Discussion

In 2009, Suissa et al. [4] found that STAF score could be used to preliminarily screen for AF among patients with ischemic stroke, and the sensitivity and specificity values of STAF scores ≥5 for AF prediction were 89% and 88%, respectively. Later studies confirmed its effectiveness [5] [13]; however, many other studies have revealed poor effectiveness [6] [7] [14]. Other scoring systems such as the LADS score and MrWALLETS score have been used to screen for AF, but their credibility and clinical values are less effective than STAF score. The current study showed that a STAF score ≥5 had a sensitivity of 78% and a specificity of 91% for AF prediction, whereas a DD level ≥ 826.5 ng/mL had a sensitivity of 70% and a specificity of 76%. When only using STAF ≥5 or DD ≥ 826.5 ng/mL for AF diagnosis, both had a poor sensitivity, and the later had a poor specificity. To enhance the accuracy of AF diagnosis, the STAF combined with DD was used, and the sensitivity and specificity of the combined methods were improved to 86% and 97%, respectively.

The items comprising the STAF score comprise the clinical data (aged and first NIHSS score) and imaging data (vascular etiology and left atrial enlargement) of the patients. It has a good screening value for AF because of the following aspects. AF primarily occurs in elderly patients, and numerous studies have confirmed that a significant correlation exists between advanced age and AF [15] [16] [17]. Relatively speaking, AF-induced cardioembolism is more severe, and the NIHSS score is higher than other types of ischemic stroke [18]. In addition, AF affects the normal systolic function of the left atrium, resulting in left atrial remodeling and leading to left atrial enlargement [19]. Left atrial enlargement can also significantly enhance the risk of AF [20]. The STAF score is primarily for patients with unknown embolic or cryptogenic ischemic stroke, and the probability of PAF in these patients is significantly higher than in those with large or small arterial disease. In summary, the STAF score can be used for AF screening; however, previous studies have also shown that the value of this score in AF screening is low. The etiological types based on TOAST have limitations. For example, some patients with ischemic stroke induced by large artery atherosclerosis have no more than 50% stenosis in the artery, and the cause of infarction might be vulnerable plaque detachment. According to STAF score, however, this group of patients scored 3 points on the last item, which significantly increased the STAF score of non-AF patients and increased the false positive rate of STAF score.

DD is one of the most important biomarkers reflecting the activation of coagulation and the fibrinolytic system. The major complication of AF is left atrial thrombosis, which leads to cerebral and peripheral thrombosis. The mechanism of thrombosis is similar to that of venous thrombosis primarily because irregular atrial contraction leads to relatively static or stagnant local blood, and the local concentrations of clotting factors (e.g., fibrinogen) increase, thereby initiating the coagulation process and promoting the formation of fibrin-rich thrombi. Thrombus occurring in the arteries have a large content of platelets, and fibrin production is secondary to platelet activation. It is possible that more DD is yielded by the degradation of emboli in the atrium after activation and secondary fibrinolysis. Therefore, the increase of DD in patients with AF combined with cerebral embolism is particularly significant [10] and can be used as one of the indicators to predict thromboembolic events in AF patients [11] [12]. Currently, no study has reported on the use of DD for AF screening. This study found that DD has certain value in the screening of AF among patients with acute ischemic stroke.

At present, approximately 1/3 of patients with ischemic stroke have diseases of unknown cause (i.e., cryptogenic stroke) [21], and PAF is associated with up to 30% of these patients. Of all of the risk factors that might cause cardioembolism, PAF is most likely missed. A Canadian study involving 17,398 ischemic stroke patients showed that 30.6% of patients underwent 24-hour Holter monitoring, and less than 1% of the patients received more than 48 hours of ECG monitoring [22]. A single 12-lead ECG or 24-hour Holter monitoring showed that the percentage of PAF in stroke patients was only 2-4%, whereas the percentage of PAF detected by 24-72 hours of ECG monitoring can be increased to 18% [23]. Furthermore, longer monitoring times are associated with higher positive rates [24]. Currently, a variety of detection methods (e.g., repeated routine ECG, Holter monitoring, continuous ECG monitoring during the hospitalization, external loop recording, and implantable loop recorder (ILR) are available). Economic analyses have suggested that long-term non-invasive ECG monitoring over 7 to 30 days is a cost-effective strategy for detecting PAF and preventing stroke recurrence [25]. Currently, however, wellestablished and reliable methods for screening patients with high-risk or suspected PAF are not available. The current study showed that STAF score combined with DD has a satisfactory screening value. This combined method can screen suspected or high-risk PAF patients, so that the aforementioned targeted examinations can be performed to eventually confirm AF and an appropriate secondary prevention program can be selected for patients with ischemic stroke. This will ultimately reduce the recurrence of stroke. Future studies with a large sample size are needed to confirm the effectiveness.

### Limitations

Due to the limited time and conditions, the limitations were inevitable. This study was a single center retrospective study of inpatients, and admission bias was inevitable. In addition, the sample size was not large, the proportion of AF patients was slightly small, and the representativeness of the sample was not so good.

## Conclusion

The STAF combined with the serum DD level has good diagnostic value in screening acute ischemic stroke patients with AF. The diagnostic sensitivity of the two indicators combined for AF was 86%, and the specificity was 97%.

## Dodatak

### Funding

This work was funded by grants from the Medical and Health Project of Science and Technology Plan of Zhuhai, China (Project No. 20191208E030032).

### Ethics approval

This study was approved by the ethics committee of Zhuhai Hospital affiliated with Jinan University. Because all patients had been discharged from the hospital, no informed consent form was signed. However, the patients' personal information was concealed to protect their privacy.

### Consent for publication

Manuscript is approved by all authors for publication.

### Acknowledgements

We thank our patients and their families. We express our sincere gratitude to all the neurologists and nurses in the Department of Neurology, Zhuhai Hospital Affiliated with Jinan University.

### Availability of data and materials

We have upload the supporting data to the attach files as an item of supplementary material.

### Conflict of interest statement

All the authors declare that they have no conflict of interest in this work.

### Contribution

Libin Liu and Peikai Xie contributed equally to this work.
